# Genome Sequence of *Bacillus simplex* Strain P558, Isolated from a Human Fecal Sample

**DOI:** 10.1128/genomeA.01241-14

**Published:** 2014-12-11

**Authors:** Olivier Croce, Perrine Hugon, Jean-Christophe Lagier, Fehmida Bibi, Catherine Robert, Esam Ibraheem Azhar, Didier Raoult, Pierre-Edouard Fournier

**Affiliations:** a Unité de Recherche sur les Maladies Infectieuses et Tropicales Emergentes, UM63, CNRS 7278, IRD 198, INSERM U1095, Faculté de Médecine, Aix-Marseille Université, Marseille, France; b Special Infectious Agents Unit, King Fahd Medical Research Center, King Abdulaziz University, Jeddah, Saudi Arabia; c Department of Medical Laboratory Technology, Faculty of Applied Medical Sciences, King Abdulaziz University, Jeddah, Saudi Arabia

## Abstract

*Bacillus simplex* strain P558 was isolated from a fecal sample of a 25-year-old Saudi male. We sequenced the 5.98-Mb genome of the strain and compared it to that of *B. simplex* strain 1NLA3E*.*

## GENOME ANNOUNCEMENT

*Bacillus simplex* was first described in 1901 by Meyer and Gottheil (1). This bacterium is an environmental microorganism, notably found in soil. To date, it has not been found in humans. Here, we sequenced the genome from *B. simplex* strain P558 that was isolated from a fecal sample of a 25-year-old Saudi male living in Jeddah, Saudi Arabia, as part of a culturomics study aiming to isolate all bacterial species present in the human gut (2). Based on the sequencing of the complete 16S rRNA gene, strain P558 was found to exhibit 99.93% sequence identity with *B. simplex* strain AM2 (GenBank accession no. JQ435679), its closest phylogenetic relative. *B. simplex* strain P558 was deposited in the CSUR collection under number CSUR P558.

We sequenced the whole genome of *B. simplex* strain P558 with the MiSeq sequencer (Illumina, San Diego, CA, USA) using a mate-pair Nextera XT sample preparation kit (Illumina) in a 2× 250-bp run. The Illumina reads were trimmed using Trimmomatic (3) and then assembled with the SPAdes software (4). The obtained contigs were combined using the SSPACE (5) and Opera (6) softwares, helped by GapFiller (7) to reduce the set. Some manual refinements using the CLC Genomics software (CLC bio, Aarhus, Denmark) improved the genome assembly quality. Overall, the draft genome of *B. simplex* strain P558 consists of 8 scaffolds and a single gap, for a total size of 5,983,568 bp and a G+C content of 40.23%. The coding DNA sequences were predicted using Prodigal (8), and functional annotation was achieved using BLAST+ (9) and HMMER3 (10) against the UniProtKB database (11). Noncoding genes and miscellaneous features were predicted using the RNAmmer (12), ARAGORN (13), Rfam (14), Pfam (15), and Infernal (16) softwares.

The genome assembly of *B. simplex* strain P558 consists of 8 contigs and contains 5,814 protein-coding genes and 169 predicted RNA genes, including 11 rRNAs (3 complete rRNA operons), 50 tRNAs, 1 transfer-messenger RNA (tmRNA), and 107 miscellaneous RNAs. The coding capacity was 4,862,007 bp (81.26% of the total genome). Among the predicted genes, 4,248 genes (73%) matched a least one sequence in the Clusters of Orthologous Groups database (17), with BLASTp default parameters. In addition, 296 (5.09%) and 900 (15.48%) genes were annotated as encoding putative and hypothetical proteins, respectively.

In comparison with the genome of *B. simplex* strain 1NLA3E (GenBank accession no. NC_021171), the phylogenetically closest available sequenced genome, strain P558, is larger (5,983,568 and 4,815,602 bp, respectively) and has a higher G+C content (40.23 and 37.95%, respectively) and more protein-coding genes (5,814 and 4,410 genes, respectively), but it has a smaller ratio of genes per Mb (972 and 1,092 genes/Mb, respectively).

### Nucleotide sequence accession numbers.

The genome sequence from *B. simplex* strain P558 has been deposited in EMBL under accession numbers CCXW01000001 to CCXW01000008.

## References

[1] Society for General Microbiology. 1989 Validation list no. 28. Int. J. Syst. Bacteriol. 39:93–94. 10.1099/00207713-39-1-93.

[2] Lagier JC Armougom F Million M Hugon P Pagnier I Robert C Bittar F Fournous G Gimenez G Maraninchi M Trape JF Koonin EV La Scola B Raoult D 2012 Microbial culturomics: paradigm shift in the human gut microbiome study. Clin. Microbiol. Infect. 18:1185–1193. 10.1111/1469-0691.12023. 23033984

[3] Bolger AM Lohse M Usadel B 2014 Trimmomatic: a flexible trimmer for Illumina sequence data. Bioinformatics 30:2114–2120. 10.1093/bioinformatics/btu170. 24695404 PMC4103590

[4] Bankevich A Nurk S Antipov D Gurevich AA Dvorkin M Kulikov AS Lesin VM Nikolenko SI Pham S Prjibelski AD Pyshkin AV Sirotkin AV Vyahhi N Tesler G Alekseyev MA Pevzner PA 2012 SPAdes: a new genome assembly algorithm and its applications to single-cell sequencing. J. Comput. Biol. 19:455–477. 10.1089/cmb.2012.0021. 22506599 PMC3342519

[5] Boetzer M Henkel CV Jansen HJ Butler D Pirovano W 2011 Scaffolding pre-assembled contigs using SSPACE. Bioinformatics 27:578–579. 10.1093/bioinformatics/btq683. 21149342

[6] Gao S Sung WK Nagarajan N 2011 Opera: reconstructing optimal genomic scaffolds with high-throughput paired-end sequences. J. Comput. Biol. 18:1681–1691. 10.1089/cmb.2011.0170. 21929371 PMC3216105

[7] Boetzer M Pirovano W 2012 Toward almost closed genomes with GapFiller. Genome Biol. 13:R56. 10.1186/gb-2012-13-6-r56. 22731987 PMC3446322

[8] Hyatt D Chen GL Locascio PF Land ML Larimer FW Hauser LJ 2010 Prodigal: prokaryotic gene recognition and translation initiation site identification. BMC Bioinformatics 11:119. 10.1186/1471-2105-11-119. 20211023 PMC2848648

[9] Camacho C Coulouris G Avagyan V Ma N Papadopoulos J Bealer K Madden TL 2009 BLAST+: architecture and applications. BMC Bioinformatics 10:421. 10.1186/1471-2105-10-421. 20003500 PMC2803857

[10] Eddy SR 2011 Accelerated profile HMM Searches. PLoS Comput. Biol. 7:e1002195. 10.1371/journal.pcbi.1002195. 22039361 PMC3197634

[11] the Uniprot Consortium 2011 Ongoing and future developments at the Universal Protein Resource. Nucleic Acids Res. 39:D214–D219. 10.1093/nar/gkq1020. 21051339 PMC3013648

[12] Lagesen K Hallin P Rødland EA Staerfeldt HH Rognes T Ussery DW 2007 RNAmmer: consistent and rapid annotation of ribosomal RNA genes. Nucleic Acids Res. 35:3100–3108. 10.1093/nar/gkm160. 17452365 PMC1888812

[13] Laslett D Canback B 2004 ARAGORN, a program to detect tRNA genes and tmRNA genes in nucleotide sequences. Nucleic Acids Res. 32:11–16. 10.1093/nar/gkh152. 14704338 PMC373265

[14] Griffiths-Jones S Bateman A Marshall M Khanna A Eddy SR 2003 Rfam: an RNA family database. Nucleic Acids Res. 31:439–441. 10.1093/nar/gkg006. 12520045 PMC165453

[15] Punta M Coggill PC Eberhardt RY Mistry J Tate J Boursnell C Pang N Forslund K Ceric G Clements J Heger A Holm L Sonnhammer EL Eddy SR Bateman A Finn RD 2012 The Pfam protein families database. Nucleic Acids Res. 40:D290–D301. 10.1093/nar/gkr1065. 22127870 PMC3245129

[16] Nawrocki EP Kolbe DL Eddy SR 2009 Infernal 1.0: inference of RNA alignments. Bioinformatics 25:1335–1337. 10.1093/bioinformatics/btp157. 19307242 PMC2732312

[17] Tatusov RL Galperin MY Natale DA Koonin EV 2000 The COG database: a tool for genomoe-scale analysis of protein functions and evolution. Nucleic Acids Res. 28:33–36. 10.1093/nar/28.1.33. 10592175 PMC102395

